# Supplementary material for: Rapid changes in Atlantic grey seal milk from birth to weaning – immune factors and indicators of metabolic strain
Source: Sci Rep. 2017 Nov 23;7:16093. doi: 10.1038/s41598-017-16187-7 (PMC5700954; doi:10.1038/s41598-017-16187-7)
Supplement: Supplementary file 1 — Supplementary information [file 41598_2017_16187_MOESM1_ESM.pdf]

Rapid changes in Atlantic grey seal milk from birth to weaning – immune factors and indicators of metabolic strain

Amanda D. Lowe[1], Sami Bawazeer [2], David G. Watson [2], Suzanne McGill [3], Richard J.S. Burchmore [3], P.P (Paddy) Pomeroy [4] and Malcolm W. Kennedy [1]

[1] Institute of Biodiversity, Animal Health and Comparative Medicine, Graham Kerr Building, College of Medical, Veterinary and Life Sciences, University of Glasgow, Glasgow G12 8QQ, Scotland, UK

[2] Strathclyde Institute of Pharmacy and Biomedical Sciences, University of Strathclyde, 161 Cathedral Street, Glasgow G4 0RE, Scotland, UK.

[3] Institute of Infection, Immunity and Inflammation and Glasgow Polyomics, College of Medical, Veterinary and Life Sciences, University of Glasgow, Garscube Campus, Glasgow G12 1QH, Scotland, UK.

[4] Sea Mammal Research Unit, Scottish Oceans Institute, University of St Andrews, St Andrews, Fife, United Kingdom.

Author for correspondence: Malcolm Kennedy (malcolm.kennedy@glasgow.ac.uk)

Supplementary information

Figure S1. The contrasting lactation times, pup growth rates, and maternal foraging or fasting times among the Pinnipedia (true seals; sea lions/eared seals/fur seals; walrus).

Figure S2. Change in appearance of grey seal milks with time after birth.

Figure S3. Depth of grey seal milk fat layer soon after birth.

Figure S4. Changing protein profiles of grey seal milk with time after birth.

Figure S5. Two-dimensional protein electrophoresis gel of Atlantic grey seal milk.

Table S1. Identification of the proteins isolated from bands excised from the 2-dimensional protein electrophoresis gels indicated in Figure S4.

Table S2. Proteins found to change in spot intensity in 2-dimensional analysis of grey seal milk sampled on days 2, 7 and 18 after birth.

Original unmodified images of the protein electrophoresis gels given in the main paper.

Identification of the two bands in the 25-30 kDa region of Figure 2, main paper, track 2.

References for Supplementary.

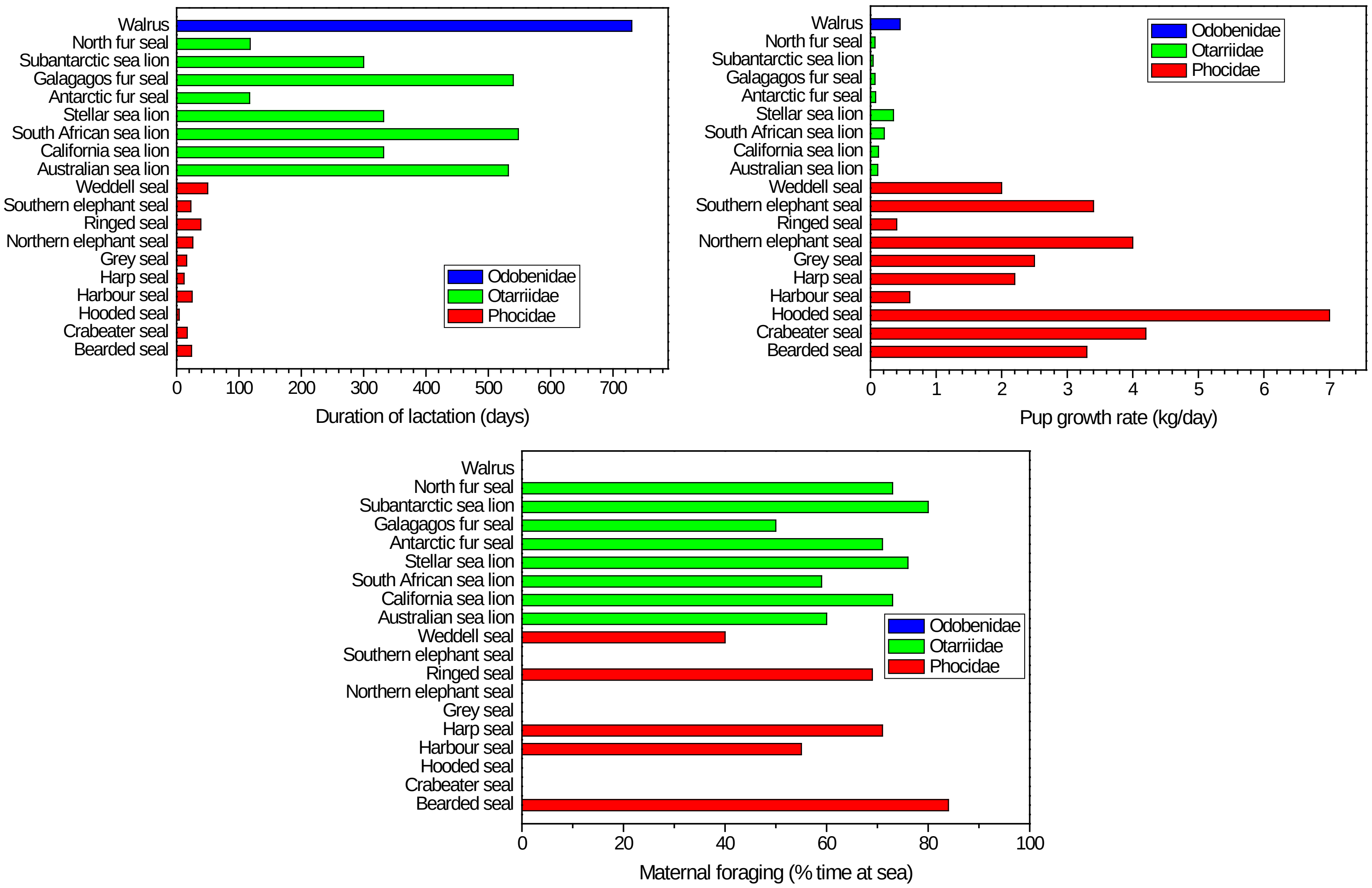

Figure S1. The contrasting lactation times, pup growth rates, and maternal foraging or fasting times among the Pinnipedia. Drawn from data taken from reference [1].

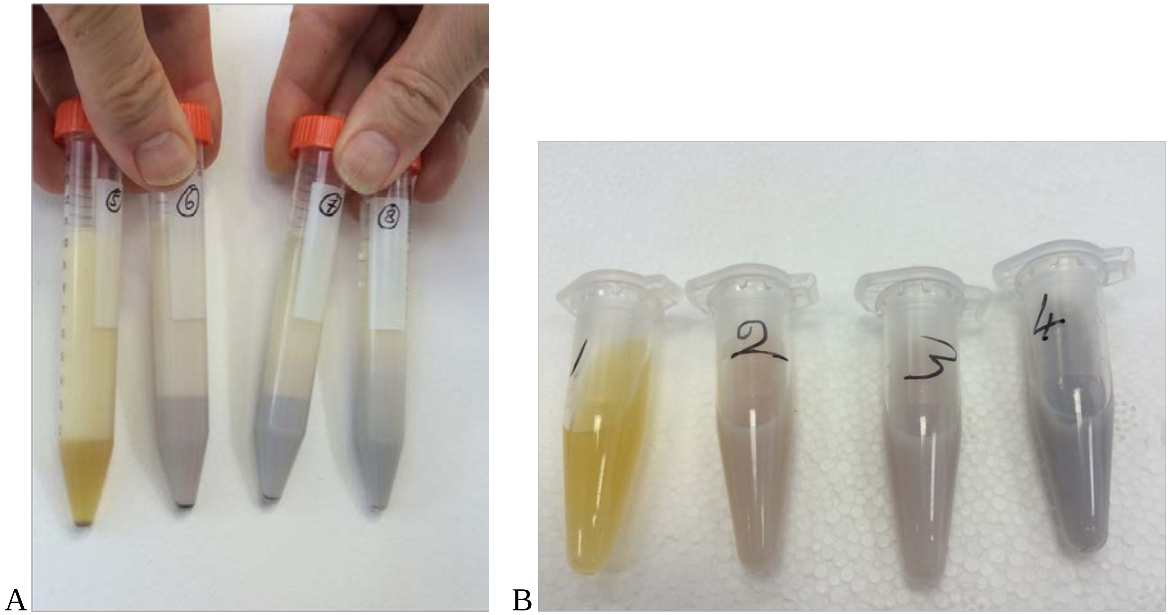

Figure S2. Change in appearance of grey seal milks with time after birth. Ten ml of each milk sample were centrifuged at 4°C. Panel A shows the resulting separation between the upper fat layers and the lower water soluble layer below. The latter layer was removed from the tubes in A to provide samples such as in panel B. The stiffness/viscosity of the fat layer decreased with duration of lactation. On top of the later samples (e.g. tube 8 in A) was a water-clear layer that was readily miscible with water; no proteins were found in this layer. The samples I panel B are from days 2, 7, 13, and 18 of an18-day lactation. The samples on the right are, in numerical order, from days 1, 7, 13, and 18 of a 21-day lactation. The images were taken in ambient laboratory lighting with a Nokia ‘smartphone’ and reproduced here with no electronic manipulation.

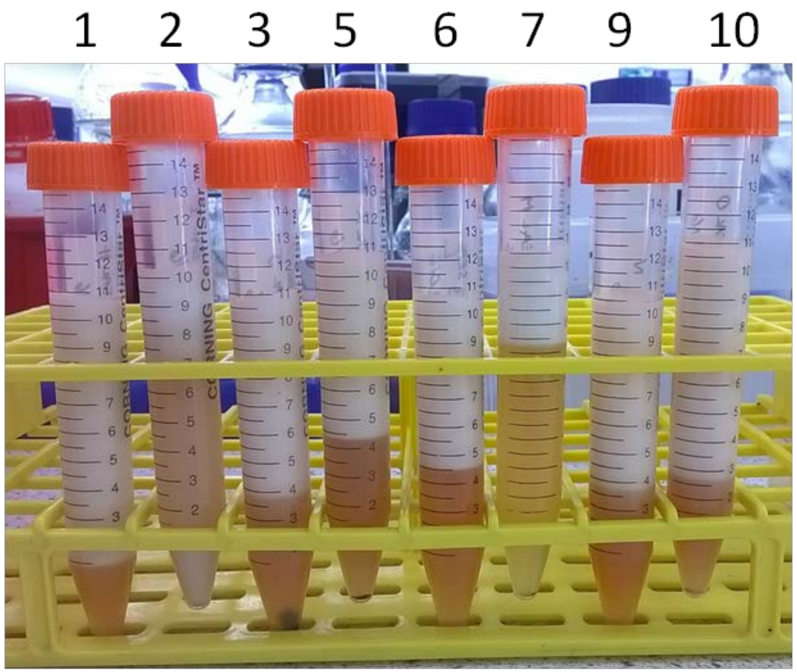

Figure S3. Depth of grey seal milk fat layer soon after birth. Milk samples were collected between 10 and 19 hours after birth and centrifuged at 4 °C as described in materials and Methods. The numbers correspond to the tracks in which these samples were analysed as seen in Figure 2 of the main text. Sample numbers 2 and 7 showed the smallest fat layers, number 2 in particular exhibiting an altered protein profile. Reduced fat content and reduced protein profile may be indicative of a colostrum phase. The images were taken in ambient laboratory lighting with a Nokia ‘smartphone’ and reproduced here with no electronic manipulation.

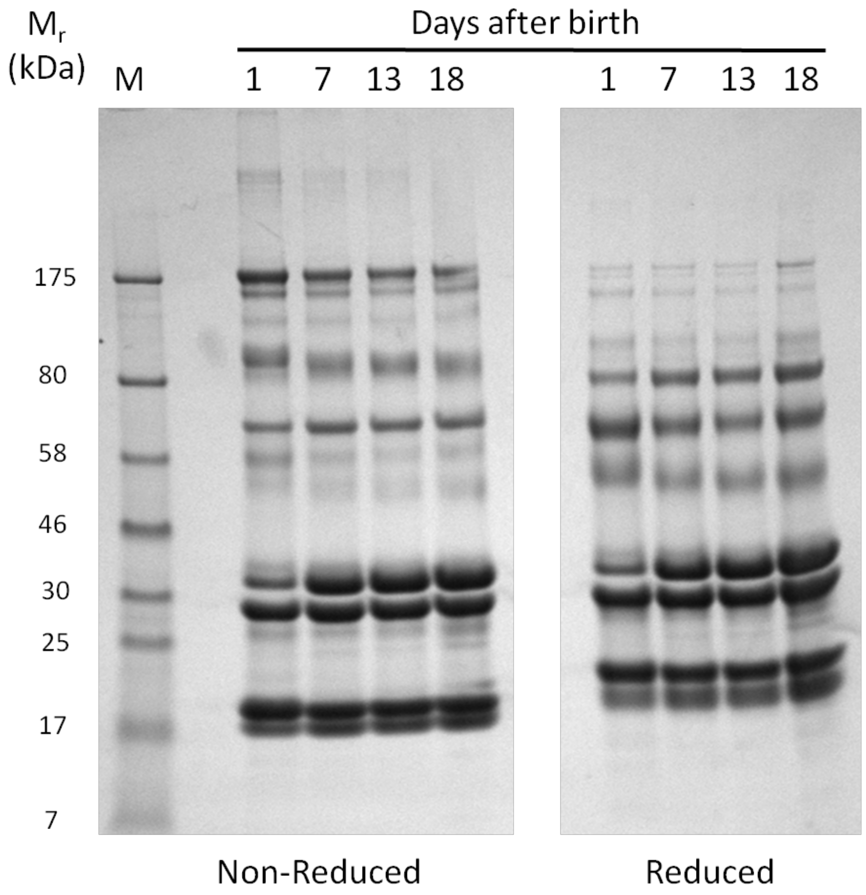

Figure S4. Changing protein profiles of grey seal milk with time after birth. Gradient SDS-PAGE of milk samples obtained from a different mother seal from that shown in Figure 1 of the main text, on the days indicated. The gel was imaged as described in Materials and Methods, and cropped to provide clarity and conciseness of presentation. Following original imaging, no further electronic manipulation was applied. The uncropped version of the gel was essentially identical to that for shown for the original image of Figure 1 of the main text shown below.

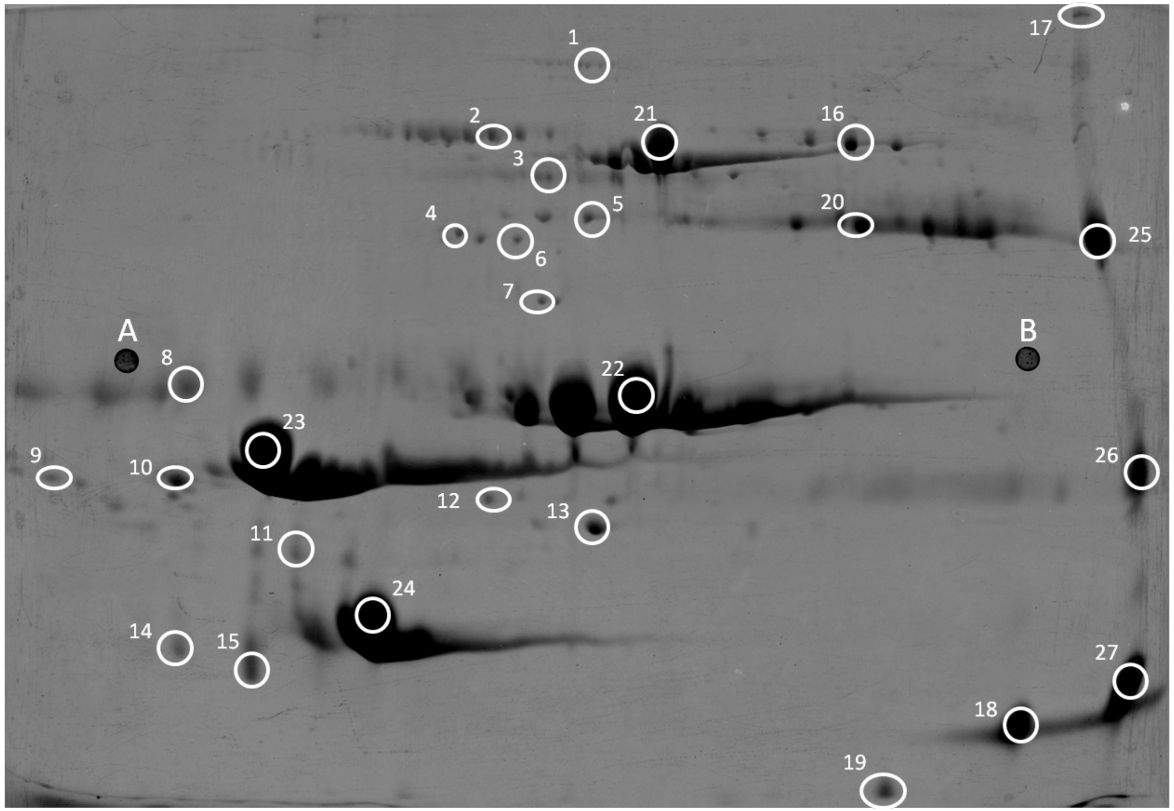

Figure S5. Two-dimensional protein electrophoresis (IEF/SDS-PAGE) gel of Atlantic grey seal milk. The sample applied to the gel was pooled from days 2, 7, and 18 for a single seal. And the numbered spots were excised and submitted to proteomics, their identities as listed in Table S1. Spots 25, 26 and 27 were to not submitted to proteomics but were inferred by position in milk samples from another member of the Caniformiae (giant panda, ref. [2]). Spots 12 and 23 contained only keratin so were excluded from Table S1. A and B are position markers, not protein spots. Two-dimensional (2-D; isoelectric focussing on a pH 4-7 immobilized pH gradient in the first dimension, SDS-PAGE on a 10% homogeneous gel in the second) gels were run using an Ettan DALT electrophoresis system (GE Healthcare, Little Chalfont Buckinghamshire HP7 9NA, UK) as previously described [2]. The stained gel was imaged as described in Materials and Methods of the main paper and elsewhere in this supplement. No post-imaging electronic manipulation was carried out.

Table S1. Identification of the proteins isolated from bands excised from the 2-dimensional protein electrophoresis gels indicated in Figure S4.

Spot number [a]	Protein indentification [b]	MASCOT score [e]	Number of peptides (unique peptide matches) [f]	Function, association, synonyms and comments [g]
1	Ceruloplasmin	76	6 (2)	The major copper-carrying protein in the blood, and plays a role in iron metabolism. Possibly involved in pulmonary antioxidant defence.
2	Polymeric immunoglobulin receptor	341	21 (6)	Receptor for IgA and IgM mediating secretion, part of which (secretory component) remains bound to IgA to protect it against proteolytic cleavage in intestine.
3	Complement component C9	97	14 (3)	Component of the membrane attack complex of the complement system. Cell lysis. Inflammation.
3	Immunoglobulin heavy chain V region	50	3 (1)	
4	Vitamin D-binding protein	451	73 (16)	Multifunctional protein found in plasma and on the surface of many cell types. Binds and stores vitamin D and its plasma metabolites and transports them to target tissues.
5	α-1-antitrypsin	188	21 (8)	Serine proteinase inhibitor. Acute-phase (fever) reactant.
6	Fetuin-B isoform X1	244	19 (7)	Proteinase inhibitor
8	κ-casein	69	6 (1)	Stabilizes micelle formation, preventing casein

				precipitation in milk.
**10**	α--casein	68	5 (0)	Important role in the transport of calcium phosphate in milk. Antioxidant peptide of it has radical scavenging activity.
**11**	β-lactoglobulin-1	133	23 (4)	Binds and probably transports retinol (vitamin A), vitamin D, and fatty acids including polyunsaturated fatty acids.
**11**	β-lactoglobulin-2	125	14 (3)	Binds and probably transports retinol (vitamin A), vitamin D, and fatty acids including polyunsaturated fatty acids.
**13**	Apolipoprotein A-I	1209	149 (46)	In plasma, transporter of cholesterol from tissues to the liver and cofactor for the lecithin cholesterol acyltransferase.
**14**	Apolipoprotein A-I	129	17 (4)	In plasma, transporter of cholesterol from tissues to the liver and cofactor for the lecithin cholesterol acyltransferase.
**14**	α-lactalbumin	91	13 (3)	Regulatory subunit of lactose synthase. Changes the substrate specificity of galactosyltransferase making glucose a good acceptor substrate for this enzyme enabling LS to synthesize lactose.
**15**	α-lactalbumin	72	21 (1)	Regulatory subunit of lactose synthase. Changes the substrate specificity of galactosyltransferase making glucose a good acceptor substrate for this enzyme enabling

				LS to synthesize lactose.
16	Bile salt-activated lipase	410	44 (13)	Presumed to assist with digestion of lipids, triglycerides in particular.
17	Immunoglobulin γ heavy chain,	83	8 (2)	
19	Fatty acid-binding protein, heart isoform	288	24 (10)	Thought to play a role in the intracellular transport of long-chain fatty acids and their acyl-CoA esters.
20	Lactadherin	531	66 (22)	Maintains intestinal epithelial homeostasis and the promotion of mucosal healing. Anti-viral.
21	Serum albumin	1691	204 (61)	Most abundant protein in blood plasma. Carries fatty acids, hydrophobic steroid hormones, hemin, small positively-charged molecules and drugs.
22	β-casein precursor	57	41 (1)	
24	β-lactoglobulin-1	547	132 (18)	Binds and probably transports retinol (vitamin A), vitamin D, and fatty acids including polyunsaturated fatty acids.
25	Immunoglobulin γ heavy chain			IgG. Antibody. Most abundant immunoglobulin class in plasma, much less so in secretions. Transferred across placenta or gut in some species by an IgG-specific receptor – situation not known in phocids.
26	Immunoglobulin κ or λ light chain			Light chain isoforms associated with all immunoglobulin subclasses.

**27**	Lysozyme C	Milk isoform of lysozyme. Anti-bacterial.

[a] Gel spot codes as indicated in Figure S4.

[b] Protein identifications. Peptides matching to keratin were excluded, as were spots for which low quality identifications were obtained.

[e] MASCOT (MOWSE) search score where scores greater than 38 are taken to be significant. The MASCOT score is the highest value obtained where the protein was identified in more than one band, as were the peptide match values.

[f] Number of peptides found to match with number of peptides unique to this identification in parentheses.

[g] Putative functions and comments are drawn from literature cited, or NCBI and UniProtKB/Swiss-Prot databases.

All proteomics data and protein diagnostics have been deposited in the Dryad Digital Repository. At the time of writing there are limited genomic, mRNA and protein sequence data available for the grey seal and the protein identifications in this table derive from searching within the Caniformia, the best fits arising from these species - *Leptonychotes weddelli* (Weddell seal), *Pusa hispida* (ringed seal), *Odobenus rosmarus divergens* (walrus), *Ailuropoda melanoleuca* (giant panda), *Mustela putorius furo* (ferret/European polecat), *Ursus maritimus* (polar bear), *Canis familiaris* (domestic dog), *Neovison vison* (American mink), *Arctocephalus tropicalis* (subantarctic fur seal). The database accession codes for the best fits are also given in the Dryad Digital Repository file. Identifications from spots 25, 26 and 27 were taken from a previous publication on milk of a Caniformian in which these bands were clearly identified [2].

Table S2. Proteins found to change in spot intensity in 2-dimensional analysis of grey seal milk sampled on days 2, 7 and 18 after birth.

Protein identification [a]	Spot number	Relative presence on day of lactation [a]	Comments [b]
Ceruloplasmin-like	1	2 > 7	
Polymeric immunoglobulin receptor	2	2 > 7	
Complement component C9	3	2 > 7	
Vitamin D-binding protein	4	2 < 7, 18	~3 fold higher level in B7 and B18 than in B2
α-1-antitrypsin	5	2 < 7 >18	~3-fold higher level in B7 than in either B2 or B18.
Fetuin-B isoform X1	6	2, 7	
Actin	7	2, 7	
Kappa-casein	8	2, 7	
α-S1-casein	10	2, 7	
β-lactoglobulin-1 & -2	11	2, 7	
Apolipoprotein A-I	13, 14	2< 7, 18	~3-fold higher level in B7 and B18 than in B2
α-lactalbumin	14, 15	2 >> 7, 18	More than 20-fold higher in B2 than B7 and B18
Bile salt-activated lipase	16	2< 7, 18	~3 fold higher level in B7 and B18 than in B2

Immunoglobulin heavy chain**	17	2	
Lysozyme C, milk isozyme	18	2, 7, 18	
Fatty acid-binding protein, heart	19	2	
Lactadherin	20	2 >> 7, 18	More than 20-fold higher in B2 than B7 and B18
Serum albumin	21	2, 7, 18	
β-casein	22		
β-lactoglobulin-1	24	2, 7, 18	
Immunoglobulin G heavy chain*	25	2, 7, 18	
Immunoglobulin light chain*	26	2, 7, 18	

[a] Proteins identified by proteomics and MASCOT searching as detailed in Table S1. All spots containing keratin, or of low confidence, were ignored.

*These proteins were not directly identified in this study but were deduced by comparison with similar analysis of giant panda milk (ref. [2]).

** M_r too high for IgG heavy chain reduced from the whole molecule. Probably therefore IgM heavy chain or unreduced IgA.

[a,b] Relative changes estimated from visual inspection and image analysis of 2-D gels (isoelectric focussing. SDS-PAGE) similar to that shown in Figure S4. 2-D difference gel electrophoresis (DIGE) was carried out as previously described [2], with 5 µl of milk samples labelled with Cy2, Cy3, and Cy5, prior to mixing and electrophoresis.

Original unmodified images of the protein electrophoresis gels presented in the main paper.

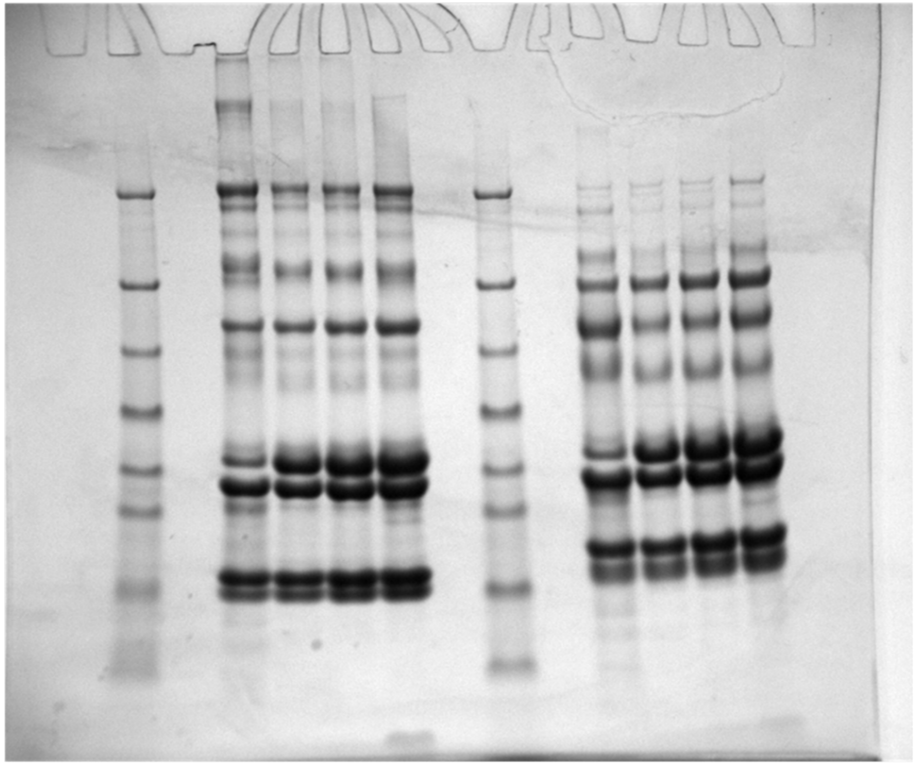

Original of Figure 1 of the main text. The complete, uncropped image of the single gel used for Figure 1 of the main text. An electronic image of the stained gel was recorded as described in Materials and Methods. As stated in the legend to Figure 1, the image was cropped and re-positioned for the final Figure, but not electronically manipulated in any way after original imaging.

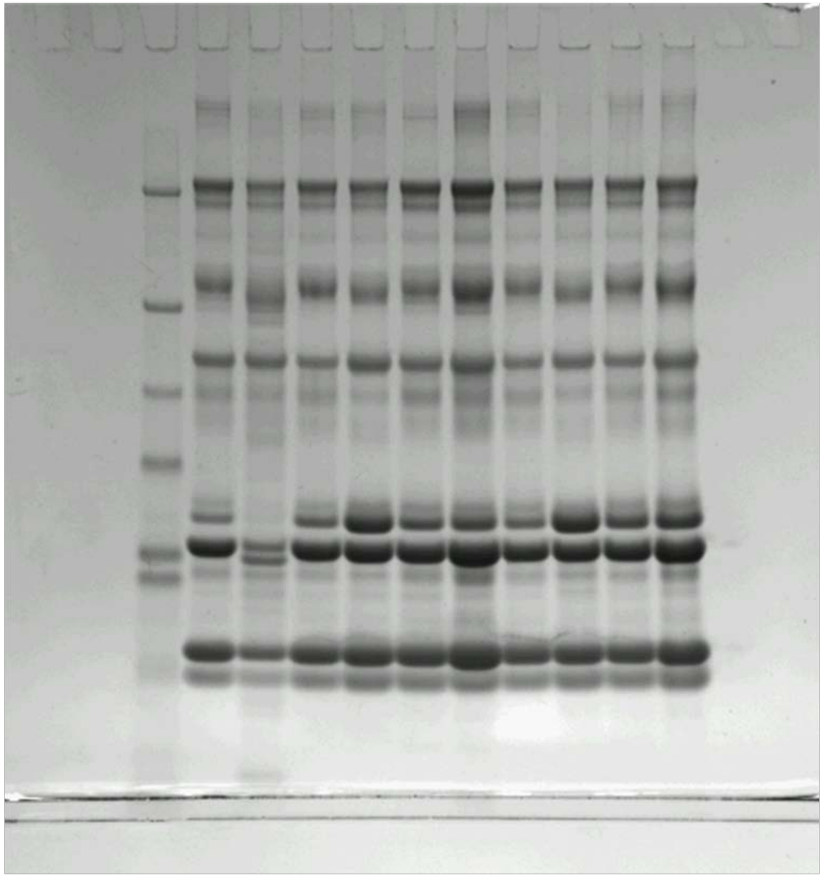

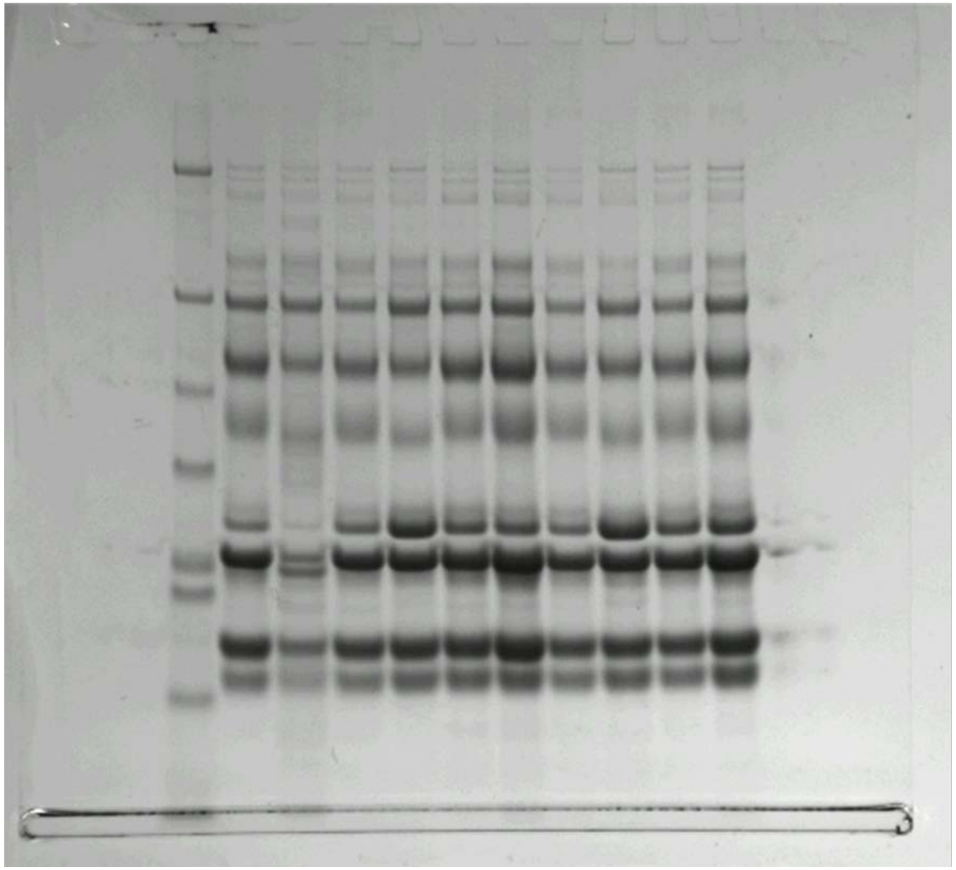

Non-reduced Reduced

Originals for Figure 2 of the main text. The complete, uncropped images of the two gels presented in Figure 2 of the main text. Electronic images of the stained gels were recorded as described in Materials and Methods. As stated in the legend to Figure 2, the images were cropped and re-positioned for the final Figure, but not electronically manipulated in any way after the original imaging.

Identification of the two bands in the 25- 30 kDa region of Figure 2, main paper, track 2 in which depletions of otherwise abundant proteins are apparent in early time points. These two protein bands were subjected to proteomic analysis as described in the main paper. The relatively low yield of protein from those bands led to us finding background signals from fragments of several proteins of greater molecular mass than commensurate with the migration of the two bands. We were nevertheless able to identify proteins that were of appropriate size. The proteins identified in the upper band (pooled between reduced and on-reducing gels) were alpha-S1 casein, phosphoglycerate mutase , immunoglobulin lambda-like polypeptide 5, and β-lactoglobulin-1-like, and in the lower band were found alpha S1 casein, immunoglobulin kappa chain, and apolipoprotein A-I. These identifications remain putative at this stage.

References for Supplementary

1 Schulz, T. M. & Bowen, W. D. Pinniped lactation strategies: Evaluation of data on maternal and offspring life history traits. *Marine Mammal Science* **20**, 86-114, doi:10.1111/j.1748-7692.2004.tb01142.x (2004).

2 Griffiths, K. *et al.* Prolonged transition time between colostrum and mature milk in a bear, the giant panda, *Ailuropoda melanoleuca*. *Royal Society Open Science* **2**, doi:10.1098/rsos.150395 (2015).
